# Germination of *Phaseolus vulgaris* L. Seeds after a Short Treatment with a Powerful RF Plasma

**DOI:** 10.3390/ijms22136672

**Published:** 2021-06-22

**Authors:** Nina Recek, Matej Holc, Alenka Vesel, Rok Zaplotnik, Peter Gselman, Miran Mozetič, Gregor Primc

**Affiliations:** 1 Jožef Stefan Institute, Jamova cesta 39, 1000 Ljubljana, Slovenia; matej.holc@ijs.si (M.H.); alenka.vesel@ijs.si (A.V.); rok.zaplotnik@ijs.si (R.Z.); miran.mozetic@ijs.si (M.M.); gregor.primc@ijs.si (G.P.); 2 Interkorn Ltd., Gančani 94, 9231 Beltinci, Slovenia; peter.gselman@interkorn.si

**Keywords:** plasma, H-mode, bean, seed, water contact angle, water uptake, radicle, germination, fungal contamination

## Abstract

Seeds of common bean (*Phaseolus vulgaris* L.), of the Etna variety, were treated with low-pressure oxygen plasma sustained by an inductively coupled radiofrequency discharge in the H-mode for a few seconds. The high-intensity treatment improved seed health in regard to fungal contamination. Additionally, it increased the wettability of the bean seeds by altering surface chemistry, as established by X-ray photoelectron spectroscopy, and increasing surface roughness, as seen with a scanning electron microscope. The water contact angle at the seed surface dropped to immeasurably low values after a second of plasma treatment. Hydrophobic recovery within a month returned those values to no more than half of the original water contact angle, even for beans treated for the shortest time (0.5 s). Increased wettability resulted in accelerated water uptake. The treatment increased the bean radicle length, which is useful for seedling establishment in the field. These findings confirm that even a brief plasma treatment is a useful technique for the disinfection and stimulation of radicle growth. The technique is scalable to large systems due to the short treatment times.

## 1. Introduction

The common bean (*Phaseolus vulgaris* L.) is an important agricultural plant grown worldwide for its seeds, also called beans. Among humans, beans are the most widely consumed legume and thus an important dietary protein source in many populations [1]. In the last two decades, the worldwide production of dry beans has nearly doubled, reaching yields close to 30 million tons annually [2]. However, the plant is commonly threatened by a variety of diseases. Like many other legumes, the common bean is severely affected by viruses and mold diseases, causing crop reduction as well as poor quality of the grains [3,4]. The mold diseases are caused by an array of fungal pathogens [5], which hinder its successful growth and reduce crop production. In fact, dry bean seeds may be naturally contaminated by fungi. These are commonly combatted with plant cultural practices, breeding for resistance, but also the use of chemical or biological agents to combat the blight [6].

In part to counter the use of environmentally harmful agents, the field of plasma agriculture has gained traction in recent years regarding both disease control and growth stimulation with the use of ecologically benign plasma technologies. While plasma is being applied in all stages of the agricultural process, seeds are a common target of plasma treatment. A recent review by Starič et al. sums up state of the art regarding both the plasma effects on seeds and the understanding of underlying mechanisms [7]. Seeds of a wide variety of plant species have been subject to plasma, among them many agriculturally important species, including the common bean.

Disinfection of seeds with plasma, particularly the removal of fungal disease agents, is common. When applied properly, the effect of plasma may reduce or eliminate the need for chemical fungicide use [8]. On the laboratory scale, this approach has been successful for many plant species, including grains such as wheat [9,10], maize [11], and rice [12], but also legumes such as soybean [13]. For bean seeds, in particular, a dielectric barrier discharge (DBD) using oxygen has prevented fungal growth after seed incubation, in which case the useful plasma treatment time was 10–30 min [14].

In addition, plasma was commonly found to stimulate several parameters of germination and growth, including germination rate and radicle length. For beans alone, several different setups of plasma reactors have positively affected seed germination and growth. Inductively coupled (IC), radio frequency (RF) air plasma has increased the germination percentage [15] and accelerated the germination [16] of bean seeds within 15 to 120 s of treatment. The seed vigor also increased through the treatment of a single seed with an argon plasma jet for 30–180 s [17]. At 5 min, the shortest treatment time in the oxygen DBD experiment, the treatment also increased hypocotyl and radicle length, but prolonged treatment inhibited germination [14]. One publication that primarily studied electrical properties in a seed-packed DBD device also found that compared to untreated seeds, plasma-treated bean seeds germinated quicker while the germination percentage remained unchanged for seeds treated for 2 min [18].

**Figure 1:**
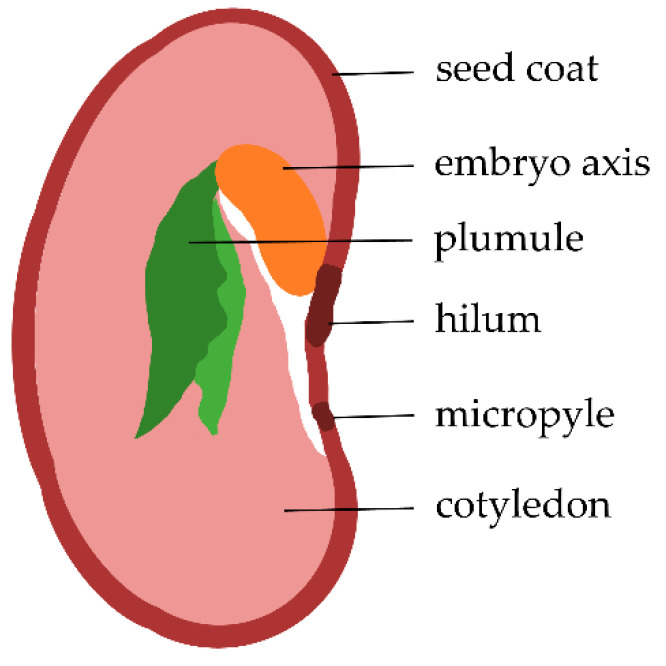
Schematic cross-section of a bean seed. Adapted from [20].

Regarding the mechanisms that lead to these biological improvements, it is advisable to start at the seed surface, where the most direct interaction between the plasma and seed takes place. To this end, let us briefly consider the botanical structure of bean seeds, as shown in Figure 1. Seeds consist of the embryo axis, which germinates into the sprout and roots, as well as the two cotyledons, which act as nutrient storage. These structures are surrounded by the seed coat [1]. The outermost layer of the seed coat—and therefore the layer directly exposed to plasma during treatment—is a protective waxy layer called the cuticle. Inwardly, the seed coat is further comprised of the epidermis, the hypodermis, and the interior parenchyma [19].

The basic structure of the seed coat, and thus of the outer layers of the seed, is very similar among the different species of the plant family *Fabaceae* [19], which includes a variety of other legumes, all related to the common bean. The most commonly encountered species with seeds used for human consumption are soybean (*Glycine max*), pea (*Pisum sativum*), lentil (*Lens culinaris*), as well as some members of the genus *Vigna*, such as mung bean (*V. radiata*), adzuki bean (*V. angularis*), and black gram (*V. mungo*). Due to the similarities, we may freely compare the surface effects of plasma on the seeds of any of these species.

Much of the currently available literature discusses the effects of plasma on the physicochemical properties of the treated seed surface. Plasma is known to alter the surface chemistry of the seeds, commonly by introducing new, oxygen-containing functional groups [15]. Additionally, it affects the morphology of the surface, often increasing the surface roughness through etching mechanisms [21]. Both of these effects result in increased wettability [15], which in turn can also lead to improved or accelerated water uptake (WU) [16].

The appearance, size, and mass of bean seeds may vary significantly between bean varieties [22]. In this work, we used seeds of the bean variety Etna. Unlike other authors, we treated them for a short time with a rather powerful low-pressure plasma. We followed its effects on the surface properties of the seeds, the wettability and WU, and ultimately, on the germination and sprout growth.

## 2. Results and Discussion

### 2.1. Water Contact Angle

**Figure 2:**
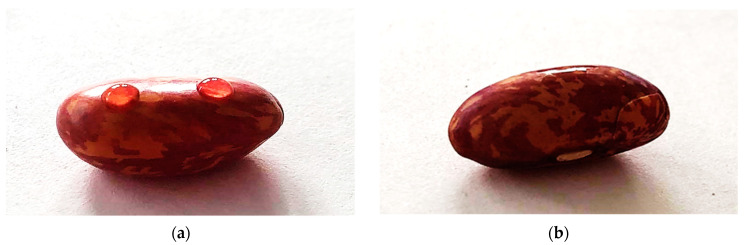
Water droplets on the surface of (**a**) an untreated bean seed and (**b**) a bean seed treated by plasma at *t* = 3 s, *p* = 10 Pa.

**Figure 3:**
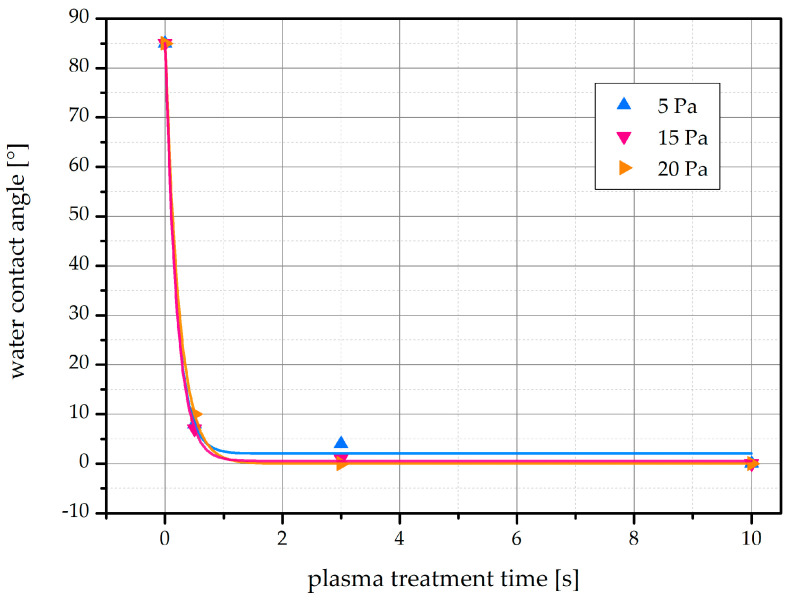
WCA on the surface of bean seeds immediately plasma treatment. Recorded after different plasma treatment times (0–10 s) using different gas pressures (5–20 Pa). Exponential curves were fitted to the data.

Water contact angles (WCA) were measured for untreated and plasma-treated samples. A comparison of the WCA for an untreated sample and a sample treated for 3 s at 10 Pa is shown in Figure 2. The WCAs immediately after plasma treatments are shown in Figure 3. The initial WCA of native bean seeds is about 85°. That value drops to about 10° after as little as 0.5 s and becomes almost immeasurably low after 3 s, indicating a super-hydrophilic surface of the seed. The effect is comparable for all gas pressures used in the experiments.

Such a rapid hydrophilization of the seed surface, as evident from Figure 3, has not been reported for legumes in scientific literature, so it is worth discussing. In two publications by Bormashenko et al., plasma treatment decreased the WCA of bean seeds from 98° to 53° within 15 s [15], and from 109° to 40° within 120 s [16]. The reactor volume was smaller in the first publication [15] but comparable to our reactor volume in the second publication [16]. While the authors have also used IC RF plasma, they ignited their air discharge at much lower nominal power of 20 W. This power would not enable ICP in the H-mode [23]. The huge difference in the WCAs after the plasma treatments can be attributed to different plasma parameters when the IC plasma is either in the E or the H-mode. Namely, the density of charged particles in the H-mode is orders of magnitude larger than in the E-mode [24,25,26]. The large flux of charged particles as well as VUV radiation [27] in the H-mode causes significant etching of the bean surface. The etching leads to rich morphology on the sub-micrometer scale, which, in combination with the polar surface functional groups, causes the super-hydrophilic surface finish. The changes to the morphology and composition of the surface after plasma treatments are presented below.

The differences in the initial WCA of untreated seeds between our measurements and those reported by Bormashenko et al. [15,16] may be attributed to biological variation between different bean varieties. Biological differences between seed species may also explain why rapid hydrophilization using E-mode RF plasma was possible in the case of wheat [15] and pepper seeds [28] at treatment times of 15 s and 5 s, respectively.

While longer treatments certainly increase the surface wettability, they may also negatively affect the ability of seeds to germinate [8]. Namely, the surface reactions triggered or caused by plasma are exothermic, so the seeds are likely to be heated upon plasma treatment. The thermal conductivity of seeds is moderate, so the interior will not be heated significantly by short plasma treatment. Since we applied H-mode plasma, where reactive species densities are high [23], short treatment times suffice to significantly alter the surface wettability without affecting the embryo temperature.

WCA after plasma treatment was also measured in other legumes. IC RF plasma reduced the WCA of lentils from 127° to 20° but did not achieve complete wetting because it was sustained in the E-mode at the same conditions (*P* = 20 W, *t* = 15 s) as for beans in the same experiment [15]. Capacitively coupled RF plasma was used for hydrophilization of mung bean and reduced its surface WCA from 100° to about 60° within 20 min of treatment [29]. However, a different capacitively coupled RF plasma reduced the WCA of soybean from 76° to 56° within 15 s [30]. The comparably smaller WCA reductions are consistent with the lower reactive species density of capacitively coupled RF plasma [23]. Among atmospheric pressure plasmas, a DBD also increased soybean wettability, but the WCA was only assessed visually. Complete wetting was not achieved within 3 min of treatment [13].

### 2.2. Hydrophobic Recovery

**Figure 4:**
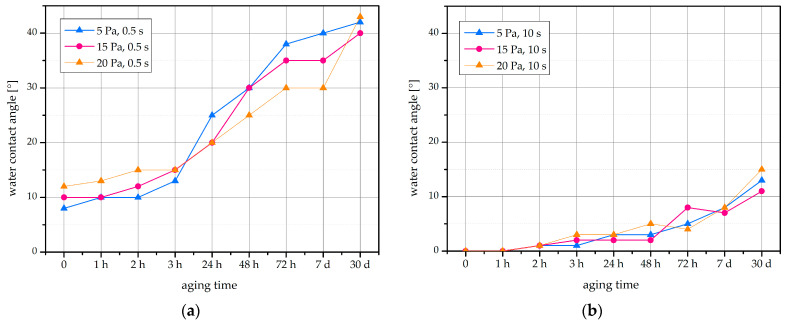
Hydrophobic recovery of the WCA on the surface of bean seeds. Recorded after plasma treatment for (**a**) 0.5 s or (**b**) 10 s at different gas pressures (5–20 Pa), followed by different times of ageing in ambient air conditions.

Hydrophobic recovery is the gradual return of the WCA to more hydrophobic values following the exposure of a hydrophilized surface to ambient conditions [31]. We followed the WCA on the bean seed surface for up to a month after plasma treatment. Results of the ageing of seeds treated for either 0.5 s or 10 s are shown in Figure 4. The shorter the plasma treatment time, the faster the WCA recovered to more hydrophobic values, and the higher the final WCA after one month of ageing at ambient air conditions. Such a dependence of the hydrophobic recovery on the plasma treatment time has been already observed for some times of the polymers [32], but the reasons are still dim. One feasible explanation is that the prolonged treatment results in a richer morphology, and consequently, a larger concentration of surface functional groups [33]. However, none of the seeds reached the initial WCA values of untreated seeds, which were about 85°. Instead, the highest WCA after one month of ageing was about 42°. For seeds exposed to plasma for longer times, the WCA increase during ageing was more gradual, and the WCA on seeds treated for 10 s reached no more than about 10–15° after a month of ageing.

While hydrophobic recovery is commonly seen with plasma-treated polymers [31], it is not well researched on seed surfaces. In fact, the available literature does not suggest an important hydrophobic recovery after the plasma treatment of seeds. In beans, along with lentils and wheat, no such effect was recorded within one month after treatment with low-pressure IC RF plasma [15], but the WCA achieved by Bormashenko et al. after the plasma treatment was as large as 53°, much larger than the WCAs reported in Figure 4.

An IC RF plasma at low power (6.8–18 W) also resulted in complete wetting of pepper seeds, a WCA reduction from 76° to immeasurably low in as little as 5 s. The authors state that no hydrophobic recovery was registered but do not specify a time frame of measurements [28]. Conversely, from an initial WCA of 100°, rice was completely hydrophilized after 30 s of treatment with a microcorona/DBD hybrid plasma, but the WCA returned to around one-third of its original value within 10 days of ageing [34]. The ageing-related decreases in surface oxygen determined by x-ray photoelectron spectroscopy (XPS) were also minor for cucumber and pepper seeds treated in an air surface DBD for 20 s and 4 s, respectively. After plasma treatment, surface oxygen increased from around 20 at.% to 40 at.% for both seed species and decreased to 35 at.% and 27 at.% during 9 days of ageing for cucumber and pepper, respectively [35].

Taking into account the above considerations, the hydrophobic recovery we see in our case may be more pronounced due to the comparably more drastic WCA decrease we achieved with our treatment.

### 2.3. Water Uptake

**Figure 5:**
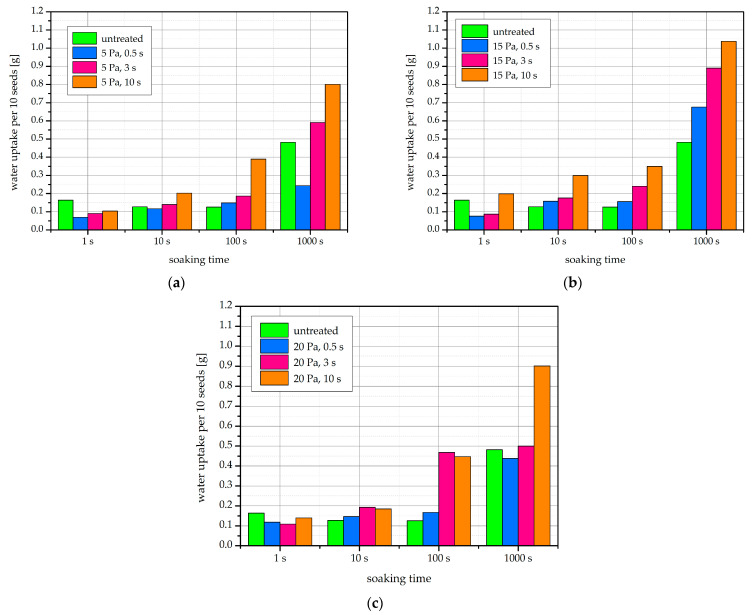
Water uptake of native and plasma-treated bean seeds expressed as g of water per 10 seeds soaked for different times (1–1000 s). Seeds were treated at (**a**) 5 Pa, (**b**) 15 Pa, and (**c**) 20 Pa.

WU for seeds soaked in water after plasma treatment is shown in Figure 5. The WU into the seeds is gradual and reaches up to about 0.8–1.0 g per 10 seed sample within 1000 s of soaking time. A trend of higher WU with longer plasma treatment time can be observed.

It has been postulated that an increase in wettability, such as that seen in Figure 3, also increases the WU by the seed [16]. This is due to the better availability of water at the seed surface, as well as the potentially increased permeability of the protective layers present at the surface. It is established that a structure called the micropyle, a small opening in the seed coat, is important for the WU of untreated bean seeds. However, it has been shown that after plasma treatment, the WU through the remaining bean seed coat surface is also strengthened [16]. Some authors suggest that the reason for the increased seed coat permeability is electroporation of the seed coat [36].

**Figure 6:**
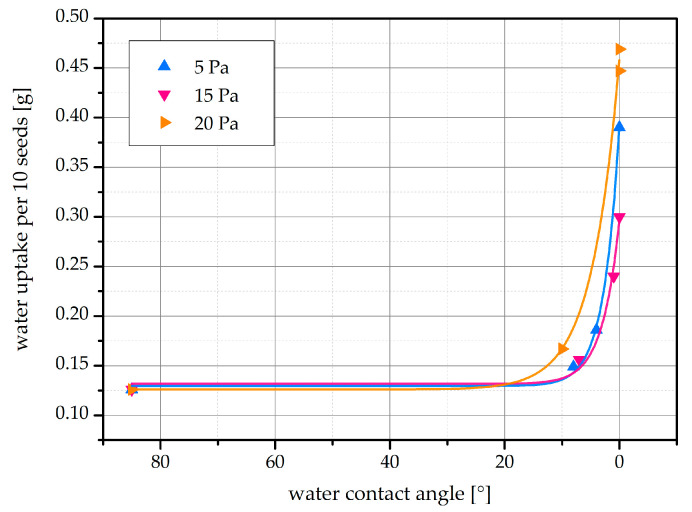
Correlation between WCA and WU for bean seeds plasma-treated at three different pressures and three different treatment times. WCA was recorded immediately after plasma treatment. WU was recorded after 100 s of soaking. Exponential curves were fitted to the data.

With soaking experiments, the link between wettability and WU increase has been previously demonstrated for beans after IC RF plasma treatment. In the two studies involving beans as well as lentils and wheat, Bormashenko et al. concluded that cold radiofrequency air plasma treatment leads to the dramatic decrease in the WCA of seeds, which in turns results in increased WU [15,16]. Further, the accumulation of increased amounts of water has been demonstrated in bean seeds by magnetic resonance imaging (MRI) following treatment with a helium plasma jet [36] and even simply after exposure to a plasma ball [37]. The correlation between WCA and WU in our own results is shown in Figure 6. The WU appears to increase exponentially with decreasing WCA. The three curves for seeds treated at different pressures nearly overlap.

Such an acceleration of WU is beneficial to the germination process, of which the water uptake by the seed is the first and essential step [38]. The presence of water allows the resumption of the biochemical processes in the seed after the metabolic quiescence during its dry state [38]. The plasma-related WU improvement can also facilitate germination and growth if the seed is planted in drought conditions [29,39].

### 2.4. Scanning Electron Microscopy

**Figure 7:**
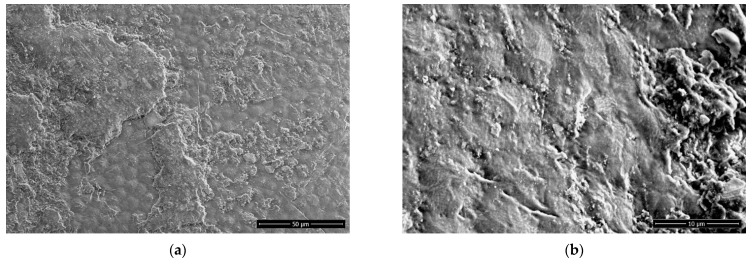

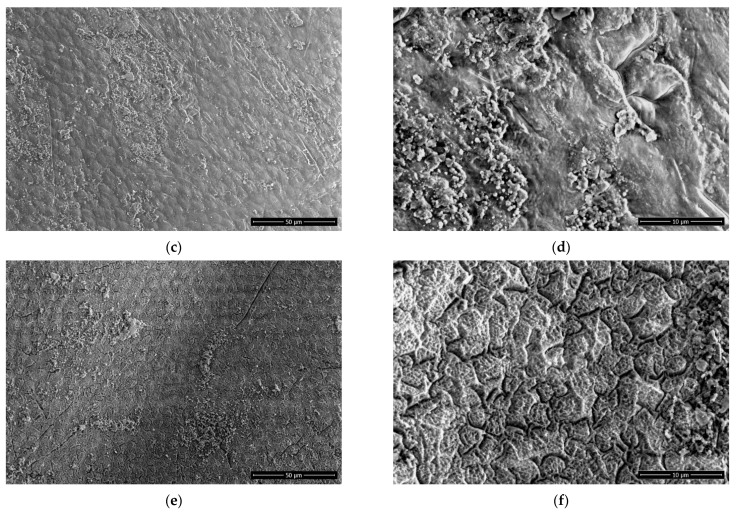
SEM images of bean surface at selected treatment conditions: (**a**,**b**) untreated; (**c**,**d**) *p* = 5 Pa, *t* = 3 s; (**e**,**f**) *p* = 20 Pa, *t* = 3 s. (**a**,**c**,**e**) 2000× magnification; (**b**,**d**,**f**) 10,000× magnification.

The obtained scanning electron microscopy (SEM) images show alterations to the bean seed surface with plasma treatment. Figure 7 shows the micrographs taken after treatment at selected conditions. At medium magnification (2000×), we mainly observe the removal of irregular materials present at the surface while the underlying scaly morphology appears to stay intact. At higher magnification (10,000×), we see that the plasma treatment alters the otherwise reasonably smooth surface significantly. The morphology becomes rougher, and particularly after the treatment at 20 Pa, structured in an apparent pattern.

The obtained micrographs assist in our understanding of the wettability and WU increase. The effect, known as plasma etching, removes material from the surface and causes increased roughness and (nano)structuring in the process. The increased surface to volume ratio contributes to increased wettability [40]. The mechanism of etching is twofold: energetic ions that impinge on the surface physically remove the material [41], while carbon atoms are also removed in volatile molecules with progressive oxidation [42]. As we know that the density of reactive plasma species in H-mode IC RF plasma is high [23], we expect considerable etching even at such short treatment times, as shown in Figure 7. The etching typically progresses in intensity with increasing treatment time [21], as the plasma removes increasing amounts of the surface material.

It has previously been shown that plasma treatment will alter the surface morphology of seeds. Interestingly, IC RF plasma treatment of beans did not change their morphology, as established by SEM [15]. As explained above, these rather marginal effects are probably due to the low power absorbed by ICP in the E-mode. As etching intensifies with increased treatment power [43], our H-mode plasma treatment causes extensive etching even though the beans are kept at a floating potential, and thus, the ion bombardment is negligible as compared to other effects. On the other hand, exposure to an oxygen DBD caused alterations to the seed coat and cotyledon surfaces, as observed by SEM at rather low magnifications [14]. Similarly, atomic force microscopy (AFM) analyses were able to detect corrugation and potentially pore formation at the bean seed surface following a helium plasma jet treatment [36]. On the other hand, the pea surface was eroded after one treatment with an air DBD [44] and showed cracks in the seed surface after a different one [21]. The surface morphology gained after plasma treatment depends on the peculiarities of the experimental reactors, so big differences between authors are expected.

### 2.5. X-ray Photoelectron Spectroscopy

The elemental composition of the bean seed surface as determined by XPS is shown in Table 1. The most apparent change after plasma treatment is the decrease in surface carbon content, accompanied by an increase in surface oxygen content to double its untreated value. The concentrations of nitrogen and silicon remain reasonably constant. After plasma treatment, additional elements appear at the seed surface, mainly magnesium and calcium, but also other elements in trace amounts.

It is well known that exposure of organic materials to oxygen-containing plasma introduces new oxygen-containing functional groups to the surface [15]. This is the reason for the drop in carbon and rise in oxygen concentration after plasma treatment. The carbon and oxygen concentrations appear quite constant regardless of the time and pressure parameters, which indicates that the surface becomes saturated with oxygen-containing functional groups already after the shortest treatment time, 0.5 s. This further indicates that the density of reactive oxygen species, particularly of the neutral oxygen atom radical, is high in this H-mode plasma, as we have previously demonstrated for this plasma system [23]. After the surface is saturated with oxygen-containing groups, etching continues, and further oxidation takes place.

Prolonged plasma treatment can etch and damage the outer layers of the plasma-treated seed and thus negatively affect it [45]. To estimate which layer of the bean seed coat the treatment effects, we can estimate the thickness of the waxy cuticle layer on the bean seed surface, which has not been assessed in the literature. One paper reports the number of cuticular alkanes on bean seeds as 3.5 mg/100 g seeds and estimates this to be about 10% of the total wax quantity [46]. We may further estimate the mass and volume of one bean seed as an average of the data given for four cultivars, which yields *m* = 0.37 g and *V* = 173.69 mm^3^, respectively [22]. If for the cuticular wax we assume a density of 1 g/cm^3^ [47], and simplify the bean seed shape to that of a sphere, we may calculate the cuticle thickness as:

(1) $$d_{\mathrm{wax}}=\sqrt[{3}]{\frac{3\;V_{\mathrm{bean}}}{4\;\pi }}-\sqrt[{3}]{\frac{3\;(V_{\mathrm{bean}}-\frac{m_{\mathrm{wax}}}{\rho_{\mathrm{wax}}})}{4\;\pi }}=0.86\;\mu m.$$

Given this thickness, along with our plasma treatment times, we expect that our plasma-mediated etching of the surface has not breached the protective layer of the bean cuticle or affected the underlying cells of the epidermis. To support this, we may consider the etching rates of polymers. We find that after 20 s of treatment with plasma, the polymers are etched at a rate no higher than 35 nm/s, with the notable exception of PVC, which structurally contains a halogen [48]. If we assume that the etching rate of cuticular wax is comparable, then our longest treatment of 10 s would etch away less than half of the total bean seed cuticle thickness.

To the best of our knowledge, this is the first XPS analysis of bean seed surface available in the literature. However, as the XPS analysis depth is on the scale of a few nm [49], we remain well within the expected cuticle thickness and may thus compare with any other seed surface XPS results. In all available analyses, XPS after plasma treatment revealed oxidation of the cuticle. Quinoa seeds treated with air plasma either as a surface DBD or low-pressure CC RF discharge [50], nasturtium seeds treated with a DBD in an air/He mixture [49], as well as cucumber and pepper seeds treated with a surface DBD in the air [35], all showed increases in surface at.% of oxygen at the expense of carbon. Analysis of high-resolution C1s XPS spectra revealed a shift from C–O bonds to O–C–O and C=O bonds and further to O–C=O bonds with treatment time increase, representative of gradual oxidation of the hydrocarbon chains. Furthermore, after surface saturation with oxygen-containing functional groups, additional oxidation results in chemical etching [49,50], which fits our SEM observations in Figure 7. As stated earlier, in the context of ageing, the concentration of oxygen at the surface may diminish with time [35]. This may be due to the re-orientation and shift of the oxygen-containing functional groups away from the surface into the bulk of the material [31].

Out of the inorganic elements, we already detected silicon on the surface of untreated beans, while magnesium, calcium, and a few minor elements appeared only after plasma treatment. These are expected elements to appear on biological sample surfaces, originating either from cellular material or environmental contamination. If we consider that after the oxidizing plasma treatment, the inorganic materials are most likely found in the form of oxides (SiO_2_, MgO, Mg(OH)_2_, CaO) [51]; the amount of oxygen taken up in this way adds up to about 5–12 at.% per treatment condition, which leaves the remaining oxygen, bonded to carbon, at about 20–30 at.%. A paper by Gómez-Ramírez et al. suggests that this accumulation of mineral elements at the seed surface after plasma treatment may be due to their inner diffusion and migration to the seed surface layers [50].

### 2.6. Germination Percentage

**Figure 8:**
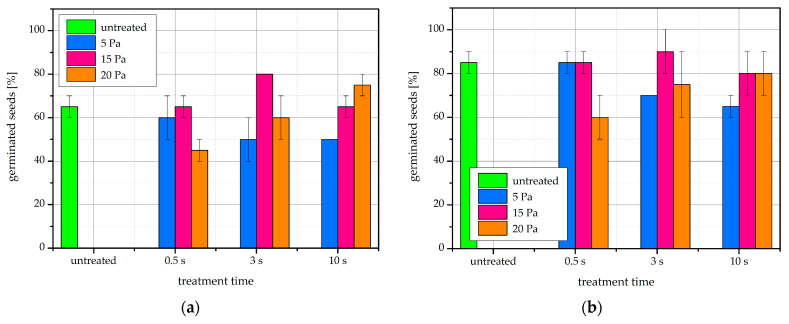
Germination percentage of native and plasma-treated bean seeds after (**a**) 4 days and (**b**) 7 days of incubation at germination conditions. The error bars represent standard error.

Within the incubation times and sample sizes used in our experiment, no notable increases in the germination percentage were recorded, as shown in Figure 8. No trends with gas pressure or treatment time were apparent. At certain treatment conditions, fewer seeds than in the untreated control germinated.

Out of the publications that have so far followed the germination percentage of bean seeds after plasma treatment, the parameter was increased by one treatment, namely an IC RF air plasma [15]. In three other cases, similarly to our results, the germination percentage was unchanged. In the case of treatment with an argon plasma jet, germination was already 100% in untreated seeds [17]. In another treatment with an IC RF air plasma [16], as well as in a seed-packed DBD reactor with helium [18], germination of untreated seeds was also >90%, and the plasma treatment accelerated the germination in both cases. Germination percentage was increased by plasma treatment in other legume species such as lentil [15], mung bean [29], pea [21], and soybean [13]. Interestingly, an early paper achieved a significant improvement in germination simply with an electrostatic treatment using a high-intensity electric field, but it does mention partial discharges between seeds, so the effect may be plasma-related [52]. If plasma treatments of seeds use power or duration that is too high, they can damage the delicate seed material [8,14,53], as the reactive species dose received by the seed is too high. It is possible that this occurred at some of the treatment conditions used in our experiments, but no correlation with treatment time nor with gas pressure is evident in Figure 8.

### 2.7. Radicle Length

**Figure 9:**
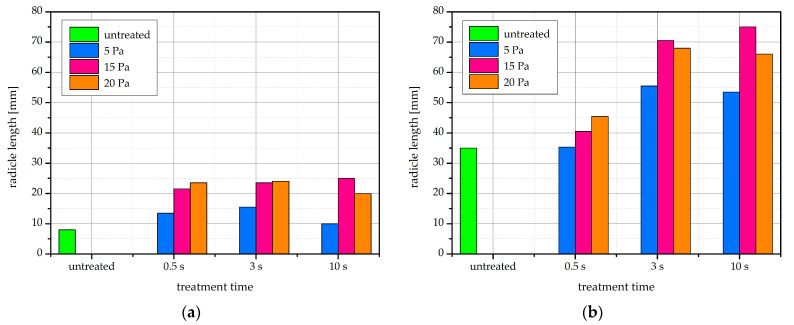
Bean seed radicle length after (**a**) 4 days and (**b**) 7 days of incubation at germination conditions.

**Figure 10:**
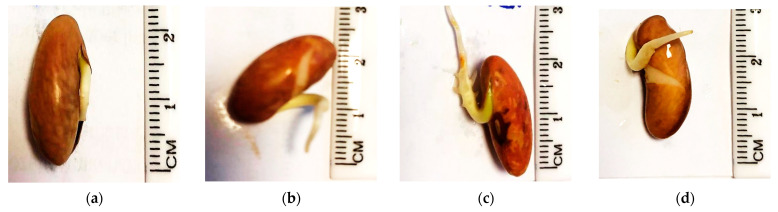
Visual comparison of bean seed radicle length after 4 days of incubation at germination conditions: (**a**) untreated; (**b**) *p* = 5 Pa, *t* = 10 s; (**c**) *p* = 15 Pa, *t* = 10 s; (**d**) *p* = 20 Pa, *t* = 10 s.

In germinating bean seeds, the first growth to protrude from the seed is the radicle, which is the embryonic root of the plant [54]. Mean radicle length after 4 and 7 days of incubation is shown in Figure 9. Radicle length increases in plasma-treated seeds compared to untreated ones. The average length of the radicle after 3 s of plasma treatment is about 55–70 mm, while it is only 35 mm for untreated beans. Further, radicle length appears to increase with plasma treatment time, at least for the first few seconds. A visual representation of radicle length after 4 days of incubation is additionally shown in Figure 10.

Volkov et al. previously found that plasma accelerates radicle development in bean seeds. This was true for seed exposure to a helium plasma jet [36] as well as a commercially available plasma ball [37]. It was also shown that the plumule, which is the seedling part that develops into the stem, increased in size after oxygen DBD treatment at a suitable treatment time of 5 min [14]. Furthermore, when the seeds were planted in soil, an increase in seedling length emerging from the soil was found after plasma exposure [36]. Seedling vigor, which is a measure of both seedling length and germination percentage, was also improved after an argon plasma jet treatment [17]. Similar improvements after plasma treatment were also observed in soybean, where one group has demonstrated that a DBD treatment of seeds may improve a variety of growth and physiology parameters in healthy seeds as well as those infected with a fungal complex [55].

Our results show that improvements in germination percentage and seedling establishment do not necessarily correlate. The radicle length improvement was achieved in the absence of a germination percentage increase. From the viewpoint of practical use in the field, an increased radicle length is beneficial for seedling establishment in the field [36].

Interestingly, one early study which placed beans into an electrostatic treatment device has achieved agricultural improvements in the absence of a discharge. The seeds, naturally infected with a bean fungus, were germinating with a higher percentage after electric-field treatment. They developed faster and had increased embryo and root mass. In the field experiments, the yield was increased by 30–80% [52]. Together with the recent results of plasma ball exposure [37], this indicates that the plasma electric field or radiation may play an important role in germination and growth stimulation. In that way, plasma exposure may be understood as a method of physical seed priming [56].

### 2.8. Fungal Contamination

**Figure 11:**
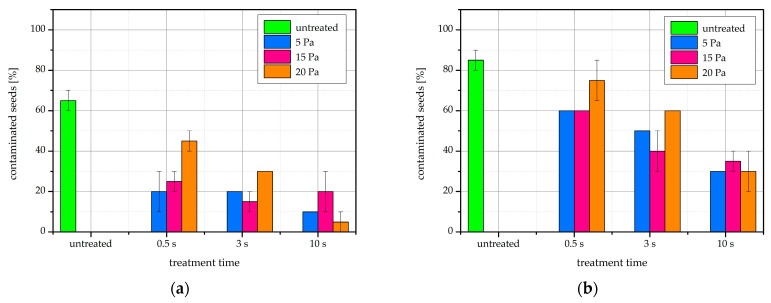
The proportion of bean seeds contaminated with a fungal infection after (**a**) 4 days and (**b**) 7 days of incubation at germination conditions. The error bars represent standard error.

**Figure 12:**
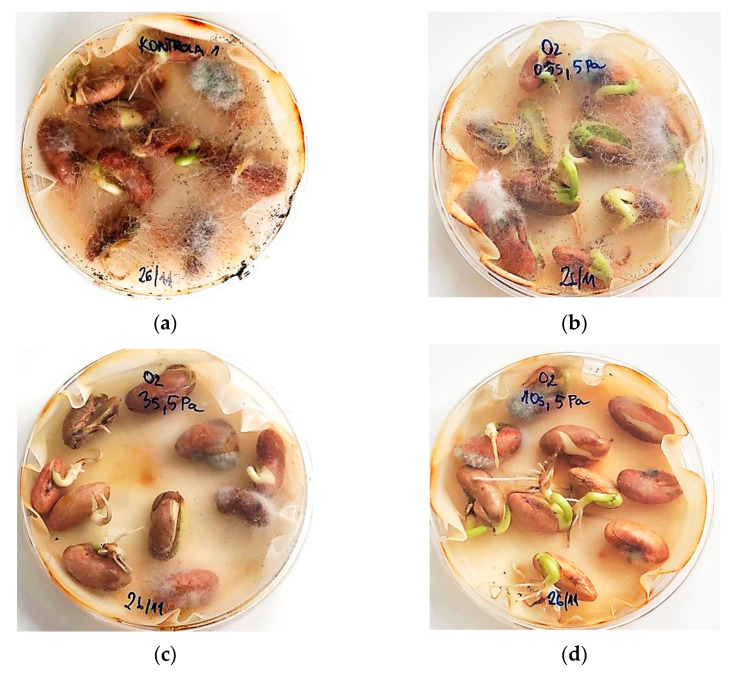
Bean seeds incubated at germination conditions after 7 days. Sprout and root growth are visible, as well as the presence of fungal contamination. (**a**) Untreated; (**b**) *p* = 5 Pa, *t* = 0.5 s; (**c**) *p* = 5 Pa, *t* = 3 s; (**d**) *p* = 5 Pa, *t* = 10 s.

During incubation of bean seeds at germination conditions, we found that seeds treated with H-mode oxygen plasma show less fungal contamination than untreated seeds. The proportion of contaminated seeds at the various treatment parameters is shown in Figure 11. The decrease in contamination compared to control is apparent and is visually illustrated in Figure 12. The percentage of infected seeds also appears to decrease with increasing treatment time. There are no clear trends regarding contamination versus gas pressure.

Bean seeds used for planting may be naturally contaminated by fungi [14,52]. A variety of fungal pathogens may attack the plant during its development and growth phases, thus preventing its successful establishment or diminishing its yield [6]. In addition, fungi can infect stored seed materials and food components and even contaminate them with mycotoxins which are harmful to human health [57]. When seeds are planted to germinate or placed in laboratory germination conditions, they require water to start this process. However, the humid environment also enables the development or spread of fungal pathogens [6]. Indeed, the moisture content of the bean seed sample itself may be high enough to allow the development of fungal spores [14]. Therefore, an environmentally friendly treatment that could remove such pathogens would be highly desired, making plasma an ideal candidate. In fact, in addition to the direct destruction of fungi, plasma has also been shown to degrade mycotoxins [58].

Results in the literature show that plasma can successfully decontaminate seeds of legumes. Bean seed decontamination has been shown in an oxygen DBD setup, where the plasma has completely inactivated the pathogens, and no fungal growth was observed in the culture. However, this occurred at treatment times already detrimental to germination [14]. In soybean infected with the seed-borne Diaporthe/Phomopsis fungal complex, DBD treatment significantly reduced the percentage of infected seeds, but the result depended on the treatment gas, time, and the dielectric material of the plasma system [13]. A surface DBD system also caused significant reductions of contamination on lentil seeds previously inoculated with fungal spores, determined using the plate count method [59]. Furthermore, on pea seeds treated with a gliding arc jet, a 60 s treatment caused a significant reduction of the microbial count [39].

The exact decontamination mechanisms of plasma technology may differ from system to system depending on plasma parameters, process gas, and consequently on the type and densities of reactive species produced [60], as well as other plasma components such as UV and/or VUV radiation [59]. When using oxygen plasma, reactive species such as the neutral oxygen atom interact with fungal spores and inactivate them as they inhibit the function of the cell membrane and oxidize the intracellular organelles [61]. In a capacitively coupled RF plasma system, the presence of oxygen and nitrogen reactive species was shown, along with the inactivation of bacteria on the cabbage seed surface [62]. Furthermore, even in the case of electrostatic treatment of beans, partial discharges between seeds were implicated in the generation of ozone and thus disinfection [52]. In IC RF plasma systems and H-mode plasmas, in particular, high electron and ion densities result in high decontamination efficiencies by the destruction of the biological contaminants [63]. Still, IC RF plasmas have only rarely been used for the treatment of seeds [15,16,28,53], and much less for seed decontamination. One such system has decreased fungal contamination in buckwheat, but the treatment also delayed germination [64].

Due to large differences in plasma types and reactor setups, it is difficult to directly compare the different plasma treatments used in the literature for seed disinfection. In other legume disinfection experiments, treatment times ranged from about a minute [13,39] to as long as 30 min [14]. Our treatment times used in this work are comparatively much shorter, up to 10 s. The difference stems from the high densities of reactive plasma species found in our H-mode IC RF plasma system [23], which destroy the fungal pathogens quickly and efficiently. Our result shows that H-mode IC RF plasma is a rapid, effective approach for removing fungi from the seed surface. If used properly, it additionally stimulates some germination and growth properties without detrimental effects on the seed.

## 3. Materials and Methods

### 3.1. Seed Material

Seeds of common bean (*Phaseolus vulgaris* L.), of the Etna variety, were obtained from Interkorn Ltd. (Gančani, Slovenia) Visually healthy and undamaged seeds were selected for the experiments. During storage, beans were kept in a dry atmosphere at room temperature to prevent early germination.

### 3.2. Plasma Reactor

**Figure 13:**
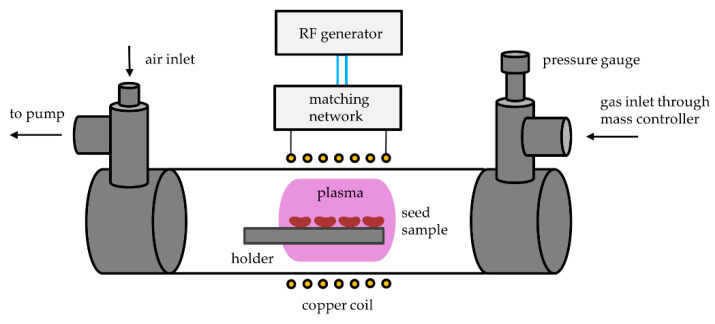
Schematic representation of the plasma system used for the treatment of bean seeds.

The reactor used for the treatment of bean seeds was an in-house, pilot-scale plasma system operating in the pressure range between about 1 and 100 Pa. Plasma was inductively coupled with an RF generator via a coil and a matching network optimized for the IC plasma in the H-mode. The glass tube of the reactor, in which seed samples are treated, was 800 mm in length and 36 mm inner diameter. The reactor was continuously pumped using a two-stage oil rotary vacuum pump with a nominal pumping speed of 80 m^3^/h. The pump was connected to the reactor on one end through a series of stainless steel tees, onto which a simple air inlet valve was mounted for venting the whole vacuum system. To achieve the desired gas pressure, commercially available oxygen of 99.99% purity was introduced at the opposite end of the tube by a flow controller AERA FC7700 (Advanced Energy, Fort Collins, CO, USA) calibrated for argon at 20 °C. The gas inlet was also connected to the reactor through a series of stainless steel tees, onto which a Baratron absolute pressure gauge (MKS Instruments, Andover, MA, USA) was mounted to monitor the pressure inside the reactor. A six-turn water-cooled copper coil with a length of 70 mm was wrapped around the tube, and a Cesar 1310 RF power generator (Advanced Energy, Fort Collins, CO, USA) operating at 13.56 MHz was coupled to the coil via a matching network. The generator operates at an adjustable output power of up to about 1 kW, but the experiments were performed at a fixed output power of 400 W. A schematic representation of the system is seen in Figure 13.

### 3.3. Plasma Treatment

During each treatment, 10 beans were treated simultaneously. The beans were placed onto an aluminum holder and positioned in the middle of the copper coil, as shown in Figure 13. The H-mode plasma is mostly spatially enclosed within the coil, so the seeds were directly exposed to the glowing plasma. The H-mode discharge was ignited at pressures ranging from 5 to 20 Pa. The output power (*P*_O_) at the generator was 400 W. An overview of treatment powers and pressures are found in Table 2. Treatment times at each gas pressure were 0.5 s, 3 s, and 10 s.

### 3.4. Water Contact Angle

We recorded the WCA on native and plasma-treated bean seeds as a measure of surface wettability. Static WCA by the sessile drop method was evaluated using the Drop Shape Analyzer DSA-100 (Krüss GmbH, Hamburg, Germany). A droplet of deionized (DI) water with a volume of V = 1 μL was placed onto a reasonably flat part of the bean seed surface. The measurements were performed at ambient conditions. For each treatment condition, WCA was measured immediately after plasma treatment, as well as after the following exposure times to ambient air: 1 h, 2 h, 3 h, 24 h, 48 h, 72 h, 7 days, and 1 month. WCA at two different positions on each seed surface was analyzed, and an average WCA from three equally treated seeds was calculated.

### 3.5. Water Uptake

To determine WU by the seeds, we submerged native and plasma-treated bean seeds into a beaker containing tap water. Soaking times were as follows: 1 s, 10 s, 100 s, and 1000 s. We calculated the mass of the seed WU by subtracting the initial dry seed mass from the seed mass after soaking. At each treatment condition, all 10 treated seeds were analyzed simultaneously. Additionally, WU was also measured and calculated 24 h, 48 h, 72 h, and 7 days after soaking.

### 3.6. Scanning Electron Microscopy

We used SEM to obtain images of the bean seed surface at high magnification (up to 10,000×). The beans were cut in half lengthwise and attached to an aluminum holder using conductive carbon tape. The SEM images were obtained using a Quanta 650 ESEM (Thermo Fisher, Waltham, MA, USA). Using an environmental SEM, there was no need for an additional coating of the sample surface.

### 3.7. X-ray Photoelectron Spectroscopy

We used an XPS instrument (Physical Electronics, Ismaning, Germany) to obtain the elemental composition of the outermost seed surface layer, expressed in atomic percent (at.%). Pieces were excised from the seed surface and placed onto the sample holder, which was placed inside the XPS and allowed to dry for an hour in the chamber before analysis. The samples were excited by X-ray radiation from a monochromatic Al source at a photon energy of 1486.6 eV. The area analyzed was 400 m^2^. The take-off angle was set at 45°. XPS survey-scan spectra were acquired at a pass-energy of 187 eV using an energy step of 0.4 eV. Spectra were analyzed using the MultiPak V8.0 software (Ulvac-phi, Inc., Chigasaki, Japan). The results for untreated and treated seeds are presented as the mean values of the analyses from two or three different bean seeds, respectively.

### 3.8. Germination and Radicle Length

We recorded the germination of bean seeds in a laboratory setting. We placed native and plasma-treated bean seeds in Petri dishes on a layer of filter paper moistened with water. The seeds were incubated at room temperature; water was added as needed. At 4 and 7 days after incubation, we counted the number of germinated seeds, i.e., seeds where the radicle has emerged through the seed coat. Per treatment condition, two parallels of 10 seeds each were used. We additionally recorded the lengths of the radicles and calculated a mean radicle length at the same incubation times, day 4 and 7.

### 3.9. Fungal Contamination

During the germination test, we recorded the presence of fungal growth on the incubated beans at 4 and 7 days after incubation. We counted the number of infected seeds where visible fungal growth was present. Per treatment condition, two parallels of 10 seeds each were used.

## 4. Conclusions

The seeds of common bean (*Phaseolus vulgaris* L.), of the Etna variety, were treated in a low-pressure plasma reactor at pressures between 5 and 20 Pa. Plasma was sustained with a well-matched radiofrequency generator operating at the standard industrial frequency of 13.56 MHz and the forward power of 400 W. The power absorbed by plasma was estimated to about 300 W. Plasma was sustained in a glass cylinder with an inner diameter of 36 mm, and the length of the glowing plasma in the H-mode was about 100 mm. The power density was, therefore, about 3 W/cm^3^. Such plasma was found useful for rapid hydrophilization of the beans’ surface. The wettability was measured with the sessile drop method. The static water contact angle of untreated seeds was about 83°. Even half a second of plasma treatment caused a drop of the WCA to about 8°, irrespective of the gas pressure in the plasma reactor. The treatment time of 3 s enabled the super-hydrophilic surface finish where the WCA was perhaps around 2–3°. The immeasurably low WCA was observed for other treatment times up to 10 s. The scanning electron microscope images revealed rich morphology on the sub-micrometer scale, which was explained by laterally inhomogeneous etching of the seed coat. As typical for all known materials with a super-hydrophilic surface finish, hydrophobic recovery was observed upon storage of plasma-treated seeds at ambient conditions. The recovery was more pronounced for samples treated for shorter times. For samples treated for 10 s, the WCA became measurable two hours after the plasma treatment. The WCA increased to 5° after storing the samples for a week and 12° after a month. The super-hydrophilic surface finish, therefore, persisted long after the plasma treatment. The water uptake also increased with increasing plasma treatment time, but the increase was not as dramatic. For example, the water uptake after soaking the seeds in distilled water increased by a factor of 2 or 3 due to the plasma treatment. The scattering of these results was particularly severe. The surface composition changed after the oxygen-plasma treatment, which is typical for surface functionalization of any organic material. Microelements also appeared in measurable amounts, indicating selective etching of organic compounds and accumulation of ashes (metal oxides) on the seed surface. The germination percentage did not change notably after any of the plasma treatments, but the radicle length a week after the plasma treatment increased by a factor of two as compared to untreated seeds. The contamination with fungi also decreased with increasing plasma treatment time, but sterility was not observed for treatment times up to 10 s. These results indicate rapid inactivation of fungal spores on the surface, but also the inability of plasma treatment to inactivate in-seed spores. The short treatment times make this technique promising for mass application in agricultural practice.

## Figures and Tables

**Table 1:** Elemental composition of native and plasma-treated bean seed surface expressed in at.%. Excluded are elements appearing in only a few analyses, mainly in amounts < 1 at.% (K, S, Al, P, F, and Cl).

*t*	*p*	C	N	O	Si	Mg	Ca
**Untreated**		75.3	4.4	17.7	2.8	/	/
**0.5 s**	**5 Pa**	50.2	3.4	36.6	2.0	2.5	2.4
	**15 Pa**	66.4	4.6	24.9	1.6	0.8	0.8
	**20 Pa**	57.6	4.9	32.0	1.7	1.3	1.4
**3 s**	**5 Pa**	49.1	4.6	35.9	1.7	2.1	2.0
	**15 Pa**	53.1	2.1	37.4	1.6	3.0	2.2
	**20 Pa**	61.4	4.6	29.2	2.5	0.8	1.1
**10 s**	**5 Pa**	49.3	3.1	38.7	2.7	2.4	2.7
	**15 Pa**	45.1	2.6	40.9	1.8	2.2	2.8
	**20 Pa**	49.4	4.5	39.0	2.9	1.6	2.1

**Table 2:** Overview of treatment powers and pressures during plasma treatment of bean seeds. *P*_O_, output power; *P*_REF_, reflected power; *P*_EFF_ effective power.

*p* (Pa)	*P*_O_ (W)	*P*_REF_ (W)	*P*_EFF_ (W)
5	400	80	320
15	400	70	330
20	400	60	340

## References

[1] Sathe S.K., Deshpande S.S., Trugo L., Finglas P.M. (2003). Beans. Encyclopedia of Food Sciences and Nutrition.

[2] Faostat Crops Data: Beans, Dry. http://www.fao.org/faostat/en/#data/QC/visualize.

[3] Hema M., Sreenivasulu P., Patil B.L., Kumar P.L., Reddy D.V. (2014). Tropical food legumes: Virus diseases of economic importance and their control. Adv. Virus Res..

[4] Marcenaro D., Valkonen J.P. (2016). Seedborne pathogenic fungi in common bean (*Phaseolus vulgaris* cv. Inta rojo) in nicaragua. PLoS ONE.

[5] Diseases of Bean (*Phaseolus Vulgaris* L.). https://www.apsnet.org/edcenter/resources/commonnames/Pages/Bean.aspx.

[6] Gupta S.K., Singh G., Singh K.P., Jahagirdar S., Sarma B.K. (2021). Major Fungal French Bean Diseases: Epidemiology and Management. Emerging Trends in Plant Pathology.

[7] Starič P., Vogel-Mikuš K., Mozetič M., Junkar I. (2020). Effects of nonthermal plasma on morphology, genetics and physiology of seeds: A review. Plants.

[8] Hoppanová L., Medvecká V., Dyblíková J., Hudecova D., Kaliňáková B., Kryštofová S., Zahoranová A. (2020). Low-temperature plasma applications in chemical fungicide treatment reduction. Acta Chim. Slovaca.

[9] Zahoranová A., Henselová M., Hudecová D., Kaliňaková B., Kováčik D., Medvecká V., Černák M. (2016). Effect of cold atmospheric pressure plasma on the wheat seedlings vigor and on the inactivation of microorganisms on the seeds surface. Plasma Chem. Plasma Process..

[10] Filatova I., Lyushkevich V., Goncharik S., Zhukovsky A., Krupenko N., Kalatskaja J. (2020). The effect of low-pressure plasma treatment of seeds on the plant resistance to pathogens and crop yields. J. Phys. D Appl. Phys..

[11] Zahoranová A., Hoppanová L., Šimončicová J., Tučeková Z., Medvecká V., Hudecová D., Kaliňáková B., Kováčik D., Černák M. (2018). Effect of cold atmospheric pressure plasma on maize seeds: Enhancement of seedlings growth and surface microorganisms inactivation. Plasma Chem. Plasma Process..

[12] Khamsen N., Onwimol D., Teerakawanich N., Dechanupaprittha S., Kanokbannakorn W., Hongesombut K., Srisonphan S. (2016). Rice (*Oryza Sativa* L.) seed sterilization and germination enhancement via atmospheric hybrid nonthermal discharge plasma. ACS Appl. Mater. Interfaces.

[13] Pérez Pizá M.C., Prevosto L., Zilli C., Cejas E., Kelly H., Balestrasse K. (2018). Effects of non-thermal plasmas on seed-borne diaporthe/phomopsis complex and germination parameters of soybean seeds. Innov. Food Sci. Emerg. Technol..

[14] Rüntzel C.L., da Silva J.R., da Silva B.A., Moecke E.S., Scussel V.M. (2019). Effect of cold plasma on black beans (*Phaseolus Vulgaris* L.), fungi inactivation and micro-structures stability. Emir. J. Food Agric..

[15] Bormashenko E., Grynyov R., Bormashenko Y., Drori E. (2012). Cold radiofrequency plasma treatment modifies wettability and germination speed of plant seeds. Sci. Rep..

[16] Bormashenko E., Shapira Y., Grynyov R., Whyman G., Bormashenko Y., Drori E. (2015). Interaction of cold radiofrequency plasma with seeds of beans (*Phaseolus Vulgaris*). J. Exp. Bot..

[17] Rodriguez-Rojas J., Barillas L., Ching-Baltodano R., Poveda-Orozco F., Vargas V.I. Low-temperature plasma irradiation to improve germination and vigor in seeds of coriandrum sativum, lycopersicon lycopersicum, phaseolus vulgaris and raphanus sativus. Proceedings of the 16th Latin American Workshop on Plasma Physics.

[18] Judee F., Dufour T. (2020). Seed-packed dielectric barrier device for plasma agriculture: Understanding its electrical properties through an equivalent electrical model. J. Appl. Phys..

[19] Souza F.H.D.D.E., Marcos-Filho J. (2001). The seed coat as a modulator of seed-environment relationships in fabaceae. Rev. Bras. Bot..

[20] The Seed of a Typical Dicotyledonous Plant: The Bean. http://www.bio.miami.edu/dana/docs/seeds.html.

[21] Stolárik T., Henselová M., Martinka M., Novák O., Zahoranová A., Černák M. (2015). Effect of low-temperature plasma on the structure of seeds, growth and metabolism of endogenous phytohormones in pea (*Pisum Sativum* L.). Plasma Chem. Plasma Process..

[22] Wani I.A., Sogi D.S., Wani A.A., Gill B.S. (2017). Physical and cooking characteristics of some indian kidney bean (*Phaseolus Vulgaris* L.) cultivars. J. Saudi Soc. Agric. Sci..

[23] Zaplotnik R., Vesel A., Mozetič M. (2011). Transition from e to h mode in inductively coupled oxygen plasma: Hysteresis and the behaviour of oxygen atom density. Europhys. Lett..

[24] Mitsui Y., Makabe T. (2021). Review and current status: E ⇌ h mode transition in low-temperature icp and related electron dynamics. Plasma Sour. Sci. Technol..

[25] Kim K.-Y., Kim K.-H., Moon J.-H., Chung C.-W. (2020). Electrical and plasma characterization of a hybrid plasma source combined with inductively coupled and capacitively coupled plasmas for o atom generation. Phys. Plasmas.

[26] Meichsner J., Wegner T. (2018). Evaluation of oxygen species during e–h transition in inductively coupled rf plasmas: Combination of experimental results with global model. Eur. Phys. J. D.

[27] Fantz U., Briefi S., Rauner D., Wünderlich D. (2016). Quantification of the vuv radiation in low pressure hydrogen and nitrogen plasmas. Plasma Sour. Sci. Technol..

[28] Shapira Y., Multanen V., Whyman G., Bormashenko Y., Chaniel G., Barkay Z., Bormashenko E. (2017). Plasma treatment switches the regime of wetting and floating of pepper seeds. Colloids Surf. B Biointerfaces.

[29] Sadhu S., Thirumdas R., Deshmukh R.R., Annapure U.S. (2017). Influence of cold plasma on the enzymatic activity in germinating mung beans (*Vigna Radiate*). LWT.

[30] Li L., Jiang J., Li J., Shen M., He X., Shao H., Dong Y. (2014). Effects of cold plasma treatment on seed germination and seedling growth of soybean. Sci. Rep..

[31] Fridman A. (2008). Plasma Chemistry.

[32] Mozetič M. (2020). Plasma-stimulated super-hydrophilic surface finish of polymers. Polymers.

[33] Vesel A., Zaplotnik R., Mozetič M., Primc G. (2021). Surface modification of ps polymer by oxygen-atom treatment from remote plasma: Initial kinetics of functional groups formation. Appl. Surf. Sci..

[34] Srisonphan S. (2018). Tuning surface wettability through hot carrier initiated impact ionization in cold plasma. ACS Appl. Mater. Interfaces.

[35] Štěpánová V., Slavíček P., Kelar J., Prášil J., Smékal M., Stupavská M., Jurmanová J., Černák M. (2018). Atmospheric pressure plasma treatment of agricultural seeds of cucumber (cucumis sativus l.) and pepper (capsicum annuum l.) with effect on reduction of diseases and germination improvement. Plasma Process. Polym..

[36] Volkov A.G., Hairston J.S., Marshall J., Bookal A., Dholichand A., Patel D. (2020). Plasma seeds: Cold plasma accelerates *Phaseolus vulgaris* seed imbibition, germination, and speed of seedling growth. Plasma Med..

[37] Volkov A.G., Bookal A., Hairston J.S., Patel D. (2021). Radio frequency plasma capacitor can increase rates of seeds imbibition, germination, and radicle growth. Funct. Plant. Biol..

[38] Taiz L., Zeiger E., Møller I.M., Murphy A. (2014). Plant Physiology and Development.

[39] Khatami S., Ahmadinia A. (2018). Increased germination and growth rates of pea and zucchini seed by fsg plasma. J. Theor. Appl. Phys..

[40] Phan L., Yoon S., Moon M.-W. (2017). Plasma-based nanostructuring of polymers: A review. Polymers.

[41] Dhayal M., Lee S.-Y., Park S.-U. (2006). Using low-pressure plasma for carthamus tinctorium l. Seed surface modification. Vacuum.

[42] Grzegorzewski F., Rohn S., Kroh L.W., Geyer M., Schlüter O. (2010). Surface morphology and chemical composition of lamb’s lettuce (*Valerianella Locusta*) after exposure to a low-pressure oxygen plasma. Food Chem..

[43] Baldanov B.B., Ranzhurov T.V., Sordonova M.N., Budazhapov L.V. (2020). Changes in the properties and surface structure of grain seeds under the influence of a glow discharge at atmospheric pressure. Plasma Phys. Rep..

[44] Gao X.T., Zhang A., Heroux P., Sand W., Sun Z.Y., Zhan J.X., Wang C.H., Hao S.Y., Li Z.Y., Li Z.Y. (2019). Effect of dielectric barrier discharge cold plasma on pea seed growth. J. Agric. Food Chem..

[45] Molina R., Lalueza A., López-Santos C., Ghobeira R., Cools P., Morent R., de Geyter N., González-Elipe A.R. (2020). Physicochemical surface analysis and germination at different irrigation conditions of dbd plasma-treated wheat seeds. Plasma Process. Polym..

[46] Mukherjee A., Sengupta A., Shaw S., Sarkar S., Pal D., Das U.K. (2020). Interrelation between surface wax alkanes from red kidney bean (*Phaseolus Vulgaris* L.) seeds and adzuki bean weevil [callosobruchus chinensis (f.)] (coleoptera: Bruchidae). Legum. Res..

[47] Zeisler-Diehl V.V., Barthlott W., Schreiber L., Wilkes H. (2018). Plant Cuticular Waxes: Composition, Function, and Interactions with Microorganisms. Hydrocarbons, Oils and Lipids: Diversity, Origin, Chemistry and Fate.

[48] Vesel A., Semenič T. (2012). Etching rates of different polymers in oxygen plasma. Mater. Tehnol..

[49] Molina R., López-Santos C., Gómez-Ramírez A., Vilchez A., Espinós J.P., González-Elipe A.R. (2018). Influence of irrigation conditions in the germination of plasma treated nasturtium seeds. Sci. Rep..

[50] Gómez-Ramírez A., López-Santos C., Cantos M., García J.L., Molina R., Cotrino J., Espinós J.P., González-Elipe A.R. (2017). Surface chemistry and germination improvement of quinoa seeds subjected to plasma activation. Sci. Rep..

[51] Varnagiris S., Vilimaite S., Mikelionyte I., Urbonavicius M., Tuckute S., Milcius D. (2020). The combination of simultaneous plasma treatment with mg nanoparticles deposition technique for better mung bean seeds germination. Processes.

[52] Morar R., Munteanu R., Simion E., Munteanu I., Dascalescu L. (1999). Electrostatic treatment of bean seeds. IEEE Trans. Ind. Appl..

[53] Lonlua R., Sarapirom S. (2019). The effect of low-pressure plasma treatment on sunflower seed germination and sprouts growth rate. J. Phys. Conf. Ser..

[54] Seed and Seedling Biology. https://extension.psu.edu/seed-and-seedling-biology.

[55] Pérez-Pizá M.C., Prevosto L., Grijalba P.E., Zilli C.G., Cejas E., Mancinelli B., Balestrasse K.B. (2019). Improvement of growth and yield of soybean plants through the application of non-thermal plasmas to seeds with different health status. Heliyon.

[56] Araújo S.d.S., Paparella S., Dondi D., Bentivoglio A., Carbonera D., Balestrazzi A. (2016). Physical methods for seed invigoration: Advantages and challenges in seed technology. Front. Plant. Sci..

[57] Varga J., Tóth B., Téren J. (2005). Mycotoxin producing fungi and mycotoxins in foods in hungary in the period 1994–2002. Acta Aliment..

[58] Hojnik N., Cvelbar U., Tavcar-Kalcher G., Walsh J.L., Krizaj I. (2017). Mycotoxin decontamination of food: Cold atmospheric pressure plasma versus classic decontamination. Toxins.

[59] Waskow A., Betschart J., Butscher D., Oberbossel G., Kloti D., Buttner-Mainik A., Adamcik J., von Rohr P.R., Schuppler M. (2018). Characterization of efficiency and mechanisms of cold atmospheric pressure plasma decontamination of seeds for sprout production. Front. Microbiol..

[60] Mandal R., Singh A., Singh A.P. (2018). Recent developments in cold plasma decontamination technology in the food industry. Trends Food Sci. Technol..

[61] Hashizume H., Ohta T., Takeda K., Ishikawa K., Hori M., Ito M. (2014). Oxidation mechanism of penicillium digitatum spores through neutral oxygen radicals. Jpn. J. Appl. Phys..

[62] Ono R., Uchida S., Hayashi N., Kosaka R., Soeda Y. (2017). Inactivation of bacteria on plant seed surface by low-pressure rf plasma using a vibrating stirring device. Vacuum.

[63] Bol’shakov A.A., Cruden B.A., Mogul R., Rao M.V.V.S., Sharma S.P., Khare B.N., Meyyappan M. (2004). Radio-frequency oxygen plasma as a sterilization source. AIAA J..

[64] Mravlje J., Regvar M., Staric P., Mozetic M., Vogel-Mikus K. (2021). Cold plasma affects germination and fungal community structure of buckwheat seeds. Plants.

